# Dilated coronary arteries in a 2-month-old with *RIT1*-associated Noonan syndrome: a case report

**DOI:** 10.1186/s12887-022-03818-w

**Published:** 2023-01-02

**Authors:** Claudia V. Aniol, Jeremy W. Prokop, Surender Rajasekaran, Spencer Pageau, Sydney K. Elizer, Elizabeth A. VanSickle, Caleb P. Bupp

**Affiliations:** 1 College of Osteopathic Medicine, Rocky Vista University, Parker, CO USA; 2 Department of Pediatrics and Human Development, College of Human Medicine, Michigan State University, Grand Rapids, MI USA; 3 Department of Pharmacology and Toxicology, College of Human Medicine, Michigan State University, East Lansing, MI USA; 4 Corewell Health Office of Research, Grand Rapids, MI USA; 5 Pediatric Critical Care Medicine, Helen DeVos Children’s Hospital, Grand Rapids, MI USA; 6 Department of Internal Medicine and Pediatrics, Helen DeVos Children’s Hospital, Grand Rapids, MI USA; 7 Medical Genetics, Corewell Health and Helen DeVos Children’s Hospital, 25 Michigan St NE, Suite 2000, Grand Rapids, MI 49503 USA

**Keywords:** Noonan syndrome, Coronary artery dilation, Case report, Heart defects, *RIT1*

## Abstract

**Background:**

Noonan Syndrome is caused by variants in a variety of genes found in the RAS/MAPK pathway. As more causative genes for Noonan Syndrome have been identified, more phenotype variability has been found, particularly congenital heart defects. Here, we report a case of dilated coronary arteries in a pediatric patient with a *RIT1* variant to add to the body of literature around this rare presentation of Noonan Syndrome.

**Case presentation:**

A 2-month-old female was admitted due to increasing coronary artery dilation and elevated inflammatory markers. Rapid whole genome sequencing was performed and a likely pathogenic *RIT1* variant was detected. This gene has been associated with a rare form of Noonan Syndrome and associated heart defects. Diagnosis of the *RIT1* variant also gave reassurance about the patient’s cardiac findings and allowed for more timely discharge as she was discharged to home the following day.

**Conclusions:**

This case highlights the importance of the association between dilated coronary arteries and Noonan syndrome and that careful cardiac screening should be advised in patients diagnosed with Noonan syndrome. In addition, this case emphasizes the importance of involvement of other subspecialities to determine a diagnosis. Through multidisciplinary medicine, the patient was able to return home in a timely manner with a diagnosis and the reassurance that despite her dilated coronary arteries and elevated inflammatory markers there was no immediate concern to her health.

## Background

Noonan syndrome (NS) is an autosomal dominant genetic condition and has a frequency of about 1 in 1000–2500 live births [1]. Most patients have dysmorphic facial features, short stature, developmental and intellectual delay, heart defects, and skeletal abnormalities [2]. Specific genes have been found to be associated with Noonan syndrome, with *PTPN11 *being the gene associated with about half of the cases, which has also previously been associated with bilateral coronary artery dilation in Noonan syndrome [3, 4]. Other genes include *SOS1, KRAS, RAF1, BRAF, MEK1, SHOC2*, and *NRAS *[3]. Rarely, variants in *RIT1* gene have also been found to cause Noonan syndrome and *RIT1 *specifically has been associated with an increased incidence of hypertrophic cardiomyopathy and perinatal abnormalities including polyhydramnios [5]. All of these genes code for proteins that are part of the RAS/MAPK pathway which is a signaling pathway involved in cell differentiation and proliferation [4–6]. Thirty percent of cases of NS still have an unexplained etiology [2]. Few cases to date have described coronary artery abnormalities in children with Noonan syndrome [7]. The most common cardiac abnormalities in Noonan syndrome are pulmonic stenosis, hypertrophic cardiomyopathy, and atrial septal defects [1]. This example helps expand the cardiac phenotypic spectrum of Noonan syndrome.

## Case presentation

**Fig. 1:**
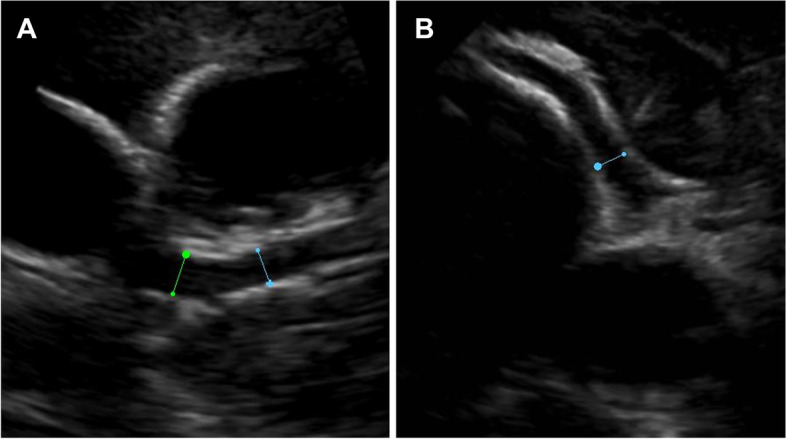
**A** Echocardiogram images at 2 months of age showing dilated coronary arteries—left main coronary artery and proximal left anterior descending and **B** right anterior descending

This patient is a 2-month-old female at the time of reporting, who was born at 36 weeks gestation via spontaneous vaginal delivery following a pregnancy complicated by polyhydramnios of unknown etiology requiring multiple amnioreductions. Genetic testing was performed on amniotic fluid and no abnormalities were found on karyotype and FISH testing. Mother was also tested and subsequently negative for infections associated with polyhydramnios. A prenatal echocardiogram showed a small muscular ventricular septal defect (VSD). Delivery and postnatal course were relatively unremarkable, however, at 1 day old, an echocardiogram was significant for a mildly dilated proximal left anterior descending artery (LAD) with a z-score of 3.3 and borderline dilation of the left main coronary artery (LMCA), with a z-score of 2.0. According to American Heart Association (AHA) guidelines, a z score ≥ 2.5 for the internal lumen diameter denotes a coronary artery abnormality [5–8]. Initially, the coronary artery dilatation was thought be related to brief runs of fetal supraventricular tachycardia, which would typically improve postnatally. The patient had frequent follow up appointments with cardiology to monitor her cardiac abnormalities. Around 2 weeks of age, she was found to have an increased gradient across her pulmonary valve (52 mmHg) and valvular dysplasia. An echocardiogram at 2 months of age showed a significant increase in coronary dilation compared to her previous echocardiograms with z-scores of proximal LAD 7.2, LMCA 3.8 and right coronary artery (RCA) 3.7 (Fig. 1). Inflammatory markers were drawn due to the concern of MIS-C or Kawasaki disease despite her appearing clinically well and were significant for a pro-BNP of 622 ng/L (5-450 ng/L), ferritin of 409 ng/mL (50-200 ng/mL), WBC of 14.23 × 10^6^/µL (6–18 × 10^3^/uL), platelets of 936 × 10^3^µL (150–450 × 10^3^/uL), erythrocyte sedimentation rate (ESR) of 8 mm/hr (0-20 mm/hr) and C-reactive protein (CRP) of 2.4 mg/L (< 5 mg/L). Given the increased z-scores and elevated pro-BNP, ferritin and platelets, she was admitted directly from the outpatient cardiology clinic for further evaluation.

On admission, the patient’s vital signs were within normal limits. Physical exam was remarkable for a female infant in no acute distress with III/VI systolic ejection murmur, with some coarse facial features, low set ears, upslanting palpebral fissures, flattened midface, sloping forehead and anteverted nares. Several more labs were obtained including IgA, IgM, IgE, C3, C4, cytokine panel and ANA; all resulted as normal. Though her mother denied recent fever, rash, conjunctival redness, and any other signs or symptoms of illness, the patient was treated for Kawasaki disease as a potential explanation for her sudden increase in coronary artery dilation. She was given one dose of IVIG 2 g/kg and was started on daily aspirin 81 mg. Roughly 13 h after her IVIG infusion completed, she became febrile to 39.5 °C. Urinalysis was collected and positive for 10 WBC/hpf, few bacteria, nitrites, and blood. Urine and blood cultures were collected. A repeat CRP was elevated at 13.8 mg/L. She was started on ceftriaxone which was discontinued when her cultures showed no growth at 48 h. Genetics was also consulted given her echocardiogram findings and dysmorphic facial features, and rapid whole genome sequencing (rWGS) was sent on day 2 of her admission. rWGS is an emerging tool that is being utilized to improve diagnostic timelines in the most at-risk populations, often diagnosing genetic conditions early enough to impact medical management. This testing was initiated due to her non-specific and complex phenotype as well as the uncertainty surrounding her hospital course. The patient underwent a coronary angiogram on day 5 of admission to better assess her coronary arteries looking for fistulas or sinusoids as well as to perform a balloon valvuloplasty of her pulmonary valve. No other cardiac abnormalities were found. Also, on day 5 of admission, rWGS preliminary results returned identifying a likely pathogenic variant in *RIT1*, a rare cause of Noonan syndrome. This diagnosis was made only 69 h after blood was drawn to initiate rWGS testing. The patient’s clinical phenotype was consistent with this diagnosis. The patient’s inflammatory markers continued to remain elevated during her inpatient stay and rWGS data was reanalyzed to ensure the patient did not have a second genetic variant causing an additional condition or syndrome. This was done using data analysis filters looking at autoimmune and inflammatory genes. The absence of additional findings on the genome provided reassurance that there was not a secondary diagnosis. Diagnosis of the *RIT1* variant also gave reassurance about the patient’s cardiac findings and allowed for more timely discharge as she was discharged to home the following day. The parents were advised to repeat labs in 1 month and follow up with Cardiology, Genetics, and Rheumatology. Subsequent testing confirmed the *RIT1* variant to be de novo, which allowed for appropriate genetic counseling for the patient’s family regarding the risk of their other children or future children also having this variant and condition. The patient is now starting to show echocardiographic evidence of evolving asymmetric hypertrophic cardiomyopathy.

## Discussion and conclusions

While *RIT1* is among the multiple genes that have been found to be associated with Noonan syndrome, variants in *RIT1* are less commonly occurring in Noonan syndrome than other identified genes. Among patients with *RIT1* gene mutation-related Noonan syndrome, cardiac deformities have been frequently reported [9], however, few cases of coronary artery abnormalities in children with Noonan syndrome have been reported, so adding another case to the body of literature is important for the medical management of these patients [7].

This case demonstrates that *RIT1*can also be associated with coronary artery dilation, however the mechanism for this finding is still undetermined. As Noonan syndrome is not a common cause of dilated coronary arteries, this case illustrates that it should be considered in the differential along with more common causes like Kawasaki disease and arteriovenous (AV) fistulas [6, 7]. This is particularly the case when there are other clinical features that may support a diagnosis of NS such as this patient’s pulmonic stenosis, polyhydramnios, and facial dysmorphisms. This patient’s inflammatory markers were also a complicating factor for diagnosis, as well as the timing of this work-up during COVID-19 pandemic where the pediatric cardiac phenotype was emerging and not well understood. From the patient’s history, she did not meet the diagnostic criteria for Kawasaki disease with no recent fever, rash, or conjunctival injection though she was still treated empirically. An AV fistula was excluded based upon cardiac catherization. Before genetic diagnosis was obtained, empiric treatment was still employed as the patient’s diagnosis was uncertain and clinically concerning.

The utilization of rWGS is also notable in this case due to the speed of diagnosis and impact on medical management. Rapid sequencing is a useful diagnostic tool that not only has been shown to improve overall care, but also to decrease the cost of that care [10–12]. It has been well studied in critical illness, but the speed of diagnosis which it offers is also amenable to inpatient care that is of lower acuity. This case demonstrates the utility of achieving a diagnosis in conjunction with the planned inpatient procedure of cardiac catheterization. Combined results explained the patient’s phenotype and provided reassurance that the patient could be safely discharged. Without rWGS results, the patient would have required longer inpatient observation before the clinical team and family would be comfortable with discharge without an explanatory diagnosis.

This case highlights the importance of the association between dilated coronary arteries and Noonan syndrome and that careful cardiac screening should be advised in patients diagnosed with Noonan syndrome. In addition, this case emphasizes the importance of involvement of other subspecialities to determine a diagnosis. Through multidisciplinary medicine and rWGS, the patient was able to achieve a clinical diagnosis in real time and return home in a timely manner with a diagnosis and the reassurance that despite her dilated coronary arteries and elevated inflammatory markers there was no immediate concern to her health.

## Data Availability

All data are available from the corresponding author on reasonable request.

## Abbreviations

AHA: American Heart Association
CRP: C-Reactive Protein
ESR: Erythrocyte Sedimentation Rate
LAD: Left Anterior Descending Artery
LMCA: Left Main Coronary Artery
RCA: Right Coronary Artery
rWGS: Rapid Whole Genome Sequencing
VSD: Ventricular Septal Defect

## References

[1] Ramond F, Duband S, Croisille P, Cavé H, Teyssier G, Adouard V (2017). Expanding the cardiac spectrum of Noonan syndrome with RIT1 variant: left main coronary artery atresia causing sudden death. Eur J Med Genet.

[2] Gos M, Fahiminiya S, Poznański J, Klapecki J, Obersztyn E, Piotrowicz M (2014). Contribution of *RIT1* mutations to the pathogenesis of Noonan syndrome: four new cases and further evidence of heterogeneity. Am J Med Genet A.

[3] Li K, Thomas MA, Haber RM (2013). Ulerythema Ophryogenes, A rarely reported cutaneous manifestation of Noonan syndrome: case report and review of the literature. J Cutan Med Surg.

[4] Gulati GS, Gupta A, Juneja R, Saxena A (2011). Ectatic coronary arteries in Noonan syndrome. Tex Heart Inst J.

[5] Yaoita M, Niihori T, Mizuno S, Okamoto N, Hayashi S, Watanabe A (2016). Spectrum of mutations and genotype–phenotype analysis in Noonan syndrome patients with *RIT1* mutations. Hum Genet.

[6] Koh AL, Tan ES, Brett MS, Lai AM, Jamuar SS, Ng I (2019). The spectrum of genetic variants and phenotypic features of Southeast Asian patients with Noonan syndrome. Mol Genet Genomic Med.

[7] Mauro DM, Flors L, Hoyer AW, Norton PT, Hagspiel KD (2016). Development of bilateral coronary artery aneurysms in a child with Noonan syndrome. Pediatr Radiol.

[8] Manlhiot C, Millar K, Golding F, McCrindle BW (2010). Improved classification of coronary artery abnormalities based only on coronary artery z-scores after Kawasaki disease. Pediatr Cardiol.

[9] Zha P, Kong Y, Wang L, Wang Y, Qing Q, Dai L (2022). Noonan syndrome caused by RIT1 gene mutation: A case report and literature review. Front Pediatr.

[10] Clark MM, Hildreth A, Baranov S, Ding Y, Chowdhury S, Watkins K (2019). Diagnosis of genetic diseases in seriously ill children by rapid whole-genome sequencing and automated phenotyping and interpretation. Sci Transl Med.

[11] Farnaes L, Hildreth A, Sweeney NM, Clark MM, Chowdhury S, Nahas S (2018). Rapid whole-genome sequencing decreases infant morbidity and cost of hospitalization. Npj Genomic Med.

[12] Kingsmore SF, Cakici JA, Clark MM, Gaughran M, Feddock M, Batalov S (2019). A randomized, controlled trial of the analytic and diagnostic performance of singleton and trio, rapid genome and exome sequencing in ill infants. Am J Hum Genet.

